# Ethical Considerations of Using ChatGPT in Health Care

**DOI:** 10.2196/48009

**Published:** 2023-08-11

**Authors:** Changyu Wang, Siru Liu, Hao Yang, Jiulin Guo, Yuxuan Wu, Jialin Liu

**Affiliations:** 1 Department of Medical Informatics West China Medical School Sichuan University Chengdu China; 2 West China College of Stomatology Sichuan University Chengdu China; 3 Department of Biomedical Informatics Vanderbilt University Medical Center Nashville, TN United States; 4 Information Center West China Hospital Sichuan University Chengdu China; 5 Department of Otolaryngology-Head and Neck Surgery West China Hospital Sichuan University Chengdu China

**Keywords:** ethics, ChatGPT, artificial intelligence, AI, large language models, health care, artificial intelligence development, development, algorithm, patient safety, patient privacy, safety, privacy

## Abstract

ChatGPT has promising applications in health care, but potential ethical issues need to be addressed proactively to prevent harm. ChatGPT presents potential ethical challenges from legal, humanistic, algorithmic, and informational perspectives. Legal ethics concerns arise from the unclear allocation of responsibility when patient harm occurs and from potential breaches of patient privacy due to data collection. Clear rules and legal boundaries are needed to properly allocate liability and protect users. Humanistic ethics concerns arise from the potential disruption of the physician-patient relationship, humanistic care, and issues of integrity. Overreliance on artificial intelligence (AI) can undermine compassion and erode trust. Transparency and disclosure of AI-generated content are critical to maintaining integrity. Algorithmic ethics raise concerns about algorithmic bias, responsibility, transparency and explainability, as well as validation and evaluation. Information ethics include data bias, validity, and effectiveness. Biased training data can lead to biased output, and overreliance on ChatGPT can reduce patient adherence and encourage self-diagnosis. Ensuring the accuracy, reliability, and validity of ChatGPT-generated content requires rigorous validation and ongoing updates based on clinical practice. To navigate the evolving ethical landscape of AI, AI in health care must adhere to the strictest ethical standards. Through comprehensive ethical guidelines, health care professionals can ensure the responsible use of ChatGPT, promote accurate and reliable information exchange, protect patient privacy, and empower patients to make informed decisions about their health care.

## Introduction

ChatGPT (OpenAI) is a large language model (LLM) and an artificial intelligence (AI) chatbot [1]. Its remarkable ability to access and analyze large amounts of information allows it to generate, categorize, and summarize text with high coherence and accuracy [2]. The user-friendly interface and remarkable features of ChatGPT have made it a preferred tool for tasks such as academic writing and examinations, garnering significant interest in the field [3-6]. Although guidelines for AI bots like ChatGPT are still being developed, ChatGPT development has exceeded initial expectations [7,8]. The latest iteration, GPT-4, surpasses ChatGPT in terms of advanced reasoning, text processing capabilities, and image analysis, and it even demonstrates a degree of “creativity” [9]. Several initiatives and organizations were working to develop such standards, including the Partnership on AI [10], the AI Now Institute [11], the European Commission’s Ethics Guidelines for Trustworthy AI [12], and AI4People [13]. Many of these have been incorporated into the practices of scientific publishers and universities, such as the Committee on Publication Ethics (COPE) [14], the International Committee of Medical Journal Editors (ICMJE) [15], and Wiley’s Research Integrity and Publishing Ethics Guidelines [16]. However, the lack of established ethical review standards for AI systems, such as ChatGPT and GPT-4, poses challenges not only in academic publishing and education but also in various fields of research.

**Figure 1:**
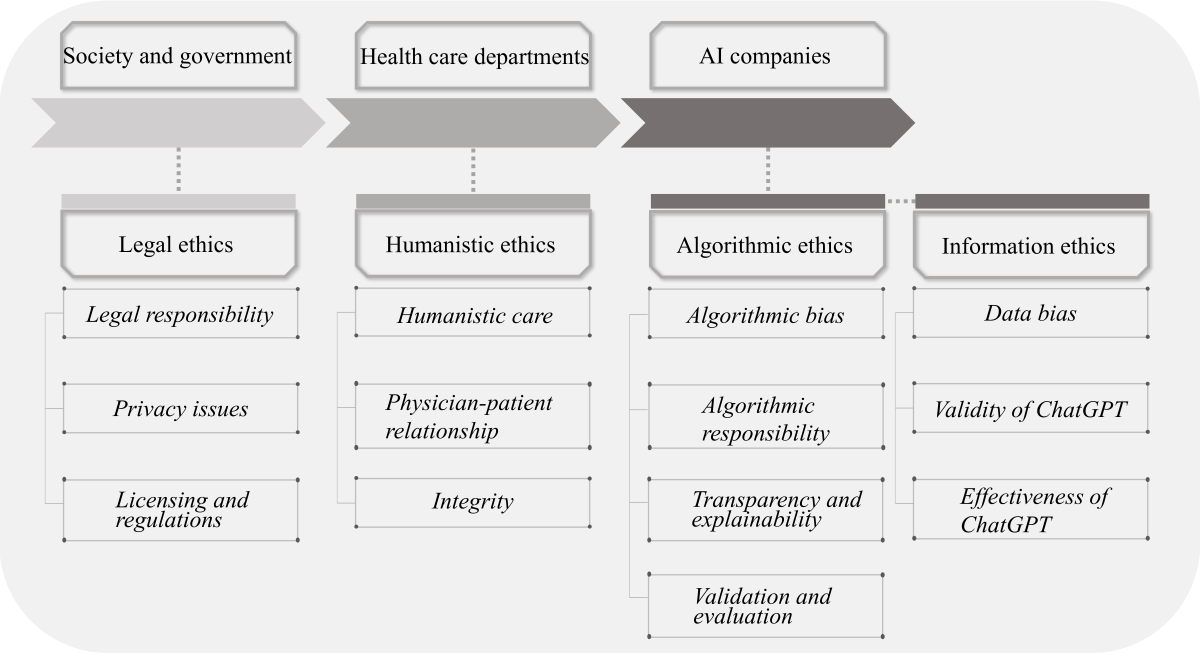
The regulatory framework of artificial intelligence (AI).

In health care, ChatGPT offers numerous benefits [17], including optimizing radiology reporting [18], generating patient discharge summaries [19], improving patient care [20], providing antimicrobial advice [21], and improving clinical decision support [22]. However, it is essential that ChatGPT adheres to principles such as beneficence, justice and fairness, medical integrity, nonmaleficence, privacy, responsibility, and transparency to prevent potential human harm [23,24]. To ensure the safe use and regulation of this technology and to facilitate public understanding, concern, and participation in the discussion, we systematically explored the potential ethical concerns associated with ChatGPT. We have considered 3 primary entities, including society and government, health care departments, and AI companies. The regulatory framework established for these 3 primary entities took into account legal ethics, humanistic ethics, algorithmic ethics, and information ethics, encompassing a total of 13 points (Figure 1) [25]. Our aim was to provide a comprehensive examination of the ethical implications of ChatGPT in health care. Given the swift advancement of AI, it is crucial to adopt a rational approach that balances the benefits and risks associated with this progress.

## Legal Ethics

### Overview

The legal ethics surrounding the use of ChatGPT in health care are an important consideration [25,26]. Essential factors to consider include the following: determining legal responsibility in cases where ChatGPT advice leads to harm or adverse outcomes; the collection and storage of sensitive patient information, which raises privacy issues; and the need to consider licensing and regulatory requirements for health care professionals when incorporating ChatGPT into clinical practice.

### Legal Responsibility

The potential for ChatGPT to provide inappropriate medical advice in real cases raises significant legal concerns [8]. From a legal perspective, AI lacks the legal status of a human being, leaving humans as the ultimate duty bearers [27]. However, determining legal responsibility in instances where a patient is harmed can indeed become a complex issue. The question arises as to who should be held accountable—the patient, the treating hospital, or OpenAI. The ambiguity underscores the need for comprehensive legal frameworks and guidelines to clearly define and allocate responsibility for the use of AI in health care.

Although OpenAI has taken steps to address these concerns by publishing detailed security standards, usage guidelines, and basic bylaws, they have also explicitly identified situations where the use of ChatGPT is prohibited. It is important to note, however, that none of these measures are currently mandatory [2]. The powerful openness of ChatGPT allows unrestricted access to all registered users. Furthermore, OpenAI explicitly disclaims any responsibility for the generated texts [28]. Consequently, it seems that the burden of any errors rests solely on the user [8]. This raises the question of who should be held responsible if inaccurate or inappropriate advice leads to harm. Clear regulations and legal restrictions are needed to properly allocate responsibility and protect users. Such regulations and laws can help establish guidelines for the use of AI systems, such as ChatGPT, in health care and outline the legal obligations of developers, health care providers, and other stakeholders.

### Privacy Issues

Privacy issues are an important aspect when using ChatGPT in health care settings [26]. The collection, storage, and processing of sensitive patient information raise important privacy issues that need to be addressed to ensure the confidentiality and protection of personal data. One of the concerns is the possibility of unauthorized access or data breaches. As ChatGPT interacts with patients and health care providers, it may gather and store personal health information. This information could encompass medical histories, test results, diagnoses, and other sensitive data. Protecting this information is critical to maintaining patient privacy and complying with applicable privacy regulations, such as the Health Insurance Portability and Accountability Act (HIPAA) in the United States or similar laws in other countries [29,30].

Another privacy concern is the risk of reidentification. Even if the data collected by ChatGPT are deidentified, there is still the potential for individuals to be reidentified by combining them with other available data sources [31]. Preventing reidentification requires strong anonymization techniques and strict access controls to prevent unauthorized linking of data.

Transparency in the use of data is also essential [32]. Patients should be informed that their data would be used by ChatGPT, and they should be given the opportunity to provide informed consent. A clear and understandable privacy policy should be in place outlining the purposes for which data are collected, where they are stored, and the measures taken to protect patient privacy [33,34].

In addition, ChatGPT’s advanced features, such as natural language processing and machine learning, may pose privacy risks [35,36]. The model may inadvertently expose sensitive information or provide inaccurate responses that could compromise patient privacy or well-being. Regular monitoring and auditing of system performance and data processing methods is essential to identify and address any privacy issues that may arise.

To mitigate privacy risks, health care organizations should implement robust security measures, including encryption, access controls, and regular vulnerability assessments [10]. A data governance framework should be in place to ensure compliance with privacy regulations and to promote responsible data handling practices. By implementing strong privacy protections and ensuring transparency and accountability in the use of ChatGPT, health care organizations can maximize the benefits of AI while protecting patient privacy and trust.

### Licensing and Regulations

As an AI-powered tool that interacts with patients and provides medical advice or support, it is necessary to implement licensing and strict regulations. This could help ChatGPT’s application in health care to meet the relevant regulatory and licensing requirements to ensure patient safety, ethical standards, and legal compliance.

Depending on the jurisdiction, health care professionals who use or rely on ChatGPT may need to hold a valid license and comply with specific regulations governing their practice. These regulations are intended to ensure that health care services provided through AI tools meet the necessary standards of care and professionalism. In addition, regulatory bodies, such as health authorities or medical boards, may need to establish guidelines or frameworks that specifically address the use of AI in health care. These guidelines could cover issues such as data privacy and security, the accuracy and reliability of AI-generated content, informed consent, and the roles and responsibilities of health care professionals when using AI tools, such as ChatGPT. Proactive and flexible regulations are necessary to ensure that AI effectively benefits patients [37]. Rigid or incomplete regulations can be detrimental and may hinder the development of AI. The current regulatory landscape for AI is still evolving [38], with ongoing efforts to establish more rigorous oversight mechanisms [39]. Although OpenAI has implemented privacy provisions and promises to handle information in an anonymized or deidentified form, the lack of sufficient regulations remains a concern [35].

Furthermore, regulatory oversight may be required to assess and approve the use of ChatGPT for specific health care applications or situations. Regulators may evaluate the safety, efficacy, and performance of AI systems, such as ChatGPT, before they are deployed in a clinical setting. This evaluation process helps to ensure that AI tools meet established standards and do not pose undue risks to patients or health care providers.

## Humanistic Ethics

### Overview

Humanistic ethics should guide the use of ChatGPT in health care, emphasizing the importance of a person-centered approach, respect for the physician-patient relationship, and integrity with patients. Health care professionals can take advantage of ChatGPT while upholding the core values of compassion, empathy, and personalized care [40,41]. Humanistic ethics in health care using ChatGPT include key aspects such as humane care, respect for the physician-patient relationship, and integrity.

### Humanistic Care

Humanistic ethics emphasize the significance of providing compassionate and individualized care [41,42]. When using ChatGPT, health care professionals should prioritize the well-being and emotional requirements of patients, ensuring that their care is not solely driven by AI-generated recommendations. Humanistic care involves offering empathetic support, actively listening to patients, and tailoring treatment plans based on a comprehensive understanding of their unique circumstances [41,43]. Although ChatGPT can provide efficient and accurate information, it lacks the human touch and empathy that are crucial in health care interactions. Health care professionals should be mindful of patients’ emotional needs and ensure that the involvement of ChatGPT does not undermine compassionate care and the overall patient experience.

### Physician-Patient Relationship

Humanistic ethics underscore the significance of the physician-patient relationship as a central component of health care [44]. When using ChatGPT, health care professionals should ensure that the presence of AI does not compromise this relationship. Health care professionals should use ChatGPT as a tool to enhance their expertise, aid in decision-making, and facilitate communication while maintaining the human connection and trust that underpin effective health care.

### Integrity

Indeed, integrity is considered a fundamental ethical principle in health care [45]. Humanistic ethics in health care using ChatGPT should involve maintaining integrity [46]. This includes transparent disclosure of AI involvement in patient care, accurate representation of ChatGPT’s limitations and capabilities, and maintaining honesty and accuracy in the communication of medical information. Health care professionals should ensure that AI-generated content, such as reports or recommendations, is based on evidence-based medicine and aligns with established clinical guidelines.

By incorporating human care, preserving the physician-patient relationship, and maintaining integrity, health care professionals can navigate the ethical implications of using ChatGPT in health care while promoting patient-centered care, empathy, and the integration of AI in a responsible and ethical manner.

## Algorithmic Ethics

### Overview

Algorithmic ethics in health care involving the use of ChatGPT require careful consideration of ethical principles and guidelines by health care professionals and organizations. This is critical to the use of ChatGPT in health care. It involves addressing the ethical implications and challenges associated with the algorithms and underlying technology that power ChatGPT. Below are some key aspects of algorithmic ethics in health care.

### Algorithmic Bias

Algorithmic bias refers to biases that arise as a result of the design, implementation, or decision-making processes within the algorithms themselves [47,48]. It occurs when the algorithms, despite being trained on unbiased data, exhibit biased behavior or produce discriminatory outcomes. Algorithmic bias can arise from several sources, including biased feature selection, biased model design, or biased decision rules [47,49]. It can amplify and exacerbate existing social, cultural, or historical biases, leading to unfair treatment or discrimination against certain individuals or groups [50-52]. In essence, data bias originates from biased data used to train the model, while algorithmic bias stems from biased decision-making processes within the model itself. Data bias can directly contribute to algorithmic bias [53], but it is possible for algorithmic bias to occur even with unbiased training data if the model’s design or decision-making mechanisms introduce bias [54,55]. This algorithmic bias could lead to clinical errors with significant consequences [56]. Even small biases in widely used algorithms can have serious consequences [57]. Concerns about the training set and underlying LLM of ChatGPT arise because OpenAI has not disclosed these details, raising suspicions that the inner workings of the AI may be hidden [58]. Besides the numerous researchers and individuals who are currently jailbreaking and testing the penetrability of generative AI, the lack of transparency in the system prevents external researchers from evaluating the bot and identifying potential algorithmic biases. It is imperative to require algorithmic transparency for all LLMs to ensure the responsible use of AI by physicians and patients. Evidence should be developed through indirect access based on results, not access to a black box. Rigorous testing and regulation of AI algorithms are necessary to protect human health [59].

### Algorithmic Responsibility

When using ChatGPT, it is important to have a clear division of responsibilities between patients, physicians, and OpenAI. Patients take responsibility for the questions they ask, ensuring that they are appropriate and relevant. Physicians, on the other hand, need to recognize and mitigate the potential “automation bias” that can result from overreliance on algorithms [60,61]. As the developer of the algorithm, OpenAI is responsible for its design and operation. It is incumbent upon OpenAI to ensure that the ChatGPT algorithm is autonomous and beneficial to patients and to justify its design choices, settings, and overall impact on society [62]. Accountability for patient protection should be rigorously enforced [63]. Health care professionals must understand their roles and responsibilities when using AI technologies such as ChatGPT and ensure that they take ultimate responsibility for decisions made based on the generated content.

### Transparency and Explainability

Transparency is a fundamental aspect of algorithmic ethics, and explainability is seen as a component of transparency [64,65]. Transparency refers to the openness and clarity of how algorithms and AI systems operate, make decisions, and generate outputs [66]. A transparent AI system provides visibility into its inner workings, enabling users and stakeholders to understand the factors that lead to its outputs. Explainability refers to the ability to provide understandable explanations for the decisions and recommendations made by an AI system [66]. Explainability is essential for users, such as health care professionals and patients, to trust AI systems and to understand the reasons behind their outputs. By ensuring transparency in algorithm ethics, AI systems like ChatGPT become more accountable and explainable. Explainability allows for the identification of potential biases, errors, or unintended consequences and facilitates the assessment of their ethical implications. In addition, transparency and interpretability help build trust and acceptance between users and stakeholders, addressing concerns about the “black box” nature of AI systems. The transparency of the algorithm enables health care professionals to comprehend how ChatGPT formulates its recommendations and allows them to explain its reasoning process to patients.

### Validation and Evaluation

Validation and evaluation are important components of algorithmic ethics [67]. They are the means by which researchers and practitioners assess the performance, accuracy, and reliability of AI algorithms [68]. Health care professionals should evaluate the accuracy, reliability, and effectiveness of the recommendations generated and compare them with established clinical guidelines and best practices. It could help promote fairness, transparency, and accountability in algorithmic decision-making, ultimately improving the quality of patient care and outcomes.

## Information Ethics

### Overview

Information ethics in health care using ChatGPT encompasses the responsible and ethical handling of data to ensure accuracy, validity, and effectiveness in the information provided by ChatGPT. It includes several key considerations.

### Data Bias

Data bias refers to the presence of biases in the training data used to develop AI models, which may not be representative of the real world or may contain systematic biases [69]. Data bias can cause AI systems to make inaccurate or unfair predictions or decisions. These biases can be unintentionally embedded in the data due to various factors, such as sampling methods, data collection processes, or human biases present in the data sources [7,70,71]. Data bias can lead to skewed outcomes and predictions, as the model learns from the biased data and perpetuates the same biases in its results [72]. AI systems, including ChatGPT, are susceptible to data bias resulting from their training data, particularly in paramedical treatments, such as developing treatment plans. AI algorithms can only generate content based on the information they have been trained on and lack the ability to generate novel ideas. If the training data used for ChatGPT are biased, the bot may inadvertently perpetuate this bias [72]. Furthermore, as the output of ChatGPT can be used to train future iterations of the model, any bias present in the data may persist without human intervention [5].

### Validity of ChatGPT

Validity refers to the accuracy, reliability, and appropriateness of the information provided by ChatGPT. As the content generated by ChatGPT can directly affect the health and well-being of patients, it is crucial to prioritize accuracy and reliability to avoid potential harm or misinformation [73]. Comprehensive validation of ChatGPT output can only be achieved through meticulous annotation of large data sets by human experts, resulting in truly valuable and reliable data [3]. However, in the case of ChatGPT, the aggregation of text and the lack of accessible source information make querying and validating responses challenging [1]. As a result, the manual validation required for ChatGPT would be extremely time and resource intensive.

Despite some limited validation efforts, ChatGPT still requires further error correction [74]. Currently, the references generated by the bot have not undergone extensive validation, leaving users to rely on their own judgment to assess the accuracy of the content [75]. This subjective assessment carries a high risk of adverse consequences. To ensure the safe and effective use of ChatGPT in health care, it is imperative that the model is trained on a substantial amount of data annotated by clinical experts and validated by physicians. This rigorous validation process could increase the reliability and trustworthiness of ChatGPT responses, ultimately benefiting patient care.

### Effectiveness of ChatGPT

Our concerns about the effectiveness of ChatGPT in health care revolve around 2 key issues: accuracy and limitations [76]. Accuracy refers to the ability of ChatGPT to generate correct and reliable information or responses in health care–related tasks. Limitations encompass the boundaries and shortcomings of ChatGPT’s capabilities, such as potential biases, lack of contextual understanding, or inability to handle complex medical scenarios.

First, ChatGPT has the potential to provide logically coherent but incorrect responses or inaccurate information due to its inability to consciously assess the accuracy of its output text [75,77]. In particular, cases of apparent error have been identified in the provision of discharge summaries and radiology reports by ChatGPT [18,19]. Therefore, clinicians should exercise caution and not overrely on ChatGPT advice, but instead, they should select clinically appropriate information.

Second, due to the nature of its training data [78], the information incorporated into ChatGPT may have delays and incompleteness [8,79]. This limitation raises concerns about its ability to provide up-to-date and comprehensive insights into the latest medical and professional research [75]. To address this, ChatGPT needs to undergo specific training and continuous updates tailored to the needs of clinical practice [73]. By moving away from fictional scenarios and focusing on providing effective answers to real health care questions [3,21], ChatGPT can increase its utility in assisting clinicians and patients.

## The Influence and Future of ChatGPT in Health Care

ChatGPT has gained widespread popularity and is beginning to reshape working practices around the world [80]. With the introduction of GPT-4, AI technology is advancing at an unprecedented rate, leading to disruptive innovation [81]. This rapid progress points to a future where AI surpasses human capabilities in processing information, including text and images. However, physicians need not resist this technological development or fear being replaced by AI [82]. In fact, the judicious use of AI can reduce procedural tasks and unleash human creativity [82]. Rather than focusing on rote learning, physicians can improve their critical thinking skills more broadly [83]. AI could be a driving force in the advancement of health care, but its integration must undergo rigorous ethical scrutiny before it is fully embraced by society.

To ensure that fundamental principles such as beneficence, nonmaleficence, medical integrity, and justice are upheld [23], AI in health care must adhere to the strictest ethical standards. Comprehensive ethical guidelines could provide essential legal, ethical, algorithmic, and informational support for patients, physicians, and health care researchers. By establishing a robust ethical framework, we can harness the potential of AI while safeguarding the well-being of individuals. This technology is expected to make a significant contribution to humanity and medicine, shaping a future where AI serves as a valuable tool to advance health care and improve patient outcomes.

## Appendix

Multimedia Appendix 1 Detailed prompts, inputs, and outputs from interaction with ChatGPT.

## Abbreviations

AI: artificial intelligence
COPE: Committee on Publication Ethics
HIPAA: Health Insurance Portability and Accountability Act
ICMJE: International Committee of Medical Journal Editors
LLM: large language model
