# Supplementary material for: Ethical Considerations of Using ChatGPT in Health Care
Source: J Med Internet Res. 2023 Aug 11;25:e48009. doi: 10.2196/48009 (PMC10457697; doi:10.2196/48009)

**Task 1**

Author’s prompt 1:

In healthcare, what ethical issues may arise from artificial intelligence?

ChatGPT (GPT-3.5) response 1:

Artificial intelligence (AI) is rapidly advancing in the healthcare industry, and while it has the potential to improve patient outcomes and increase efficiency, it also presents ethical challenges. Some of the ethical issues that may arise from the use of AI in healthcare include:

1. Bias: AI algorithms may contain bias, whether due to the data used to train the model or the way it's programmed. This could result in discrimination against certain patient populations or inaccurate diagnoses and treatments.
2. Privacy and security: AI systems may have access to large amounts of sensitive patient data, which raises concerns about privacy and security. If the data is not properly secured, it could be at risk of theft or misuse.
3. Autonomy: As AI systems become more sophisticated, they may begin to make decisions about patient care without human input. This raises questions about patient autonomy and the role of healthcare providers in the decision-making process.
4. Responsibility: If an AI system makes a mistake, who is responsible? This raises questions about liability and accountability, especially if the mistake results in harm to a patient.
5. Transparency: AI algorithms can be complex and difficult to understand, which raises questions about transparency. Patients and healthcare providers may not fully understand how the AI system is making decisions, which could erode trust in the technology.

Overall, the ethical issues surrounding AI in healthcare are complex and require careful consideration. It is essential to ensure that AI is developed and used in a way that prioritizes patient safety, privacy, and autonomy.

ChatGPT (GPT-4) response 1:

In healthcare, the integration of artificial intelligence (AI) has the potential to revolutionize diagnosis, treatment, and management of various conditions. However, this rapid growth also raises several ethical issues that need to be carefully considered:

1. Data privacy and security: Patient data is extremely sensitive and can be misused if not protected properly. Ensuring privacy, security, and compliance with data protection regulations, such as HIPAA and GDPR, is crucial when using AI in healthcare.
2. Bias and discrimination: AI systems learn from the data provided, which can perpetuate existing biases and result in unfair treatment. It is essential to monitor and minimize these biases in training datasets and algorithms to ensure equitable healthcare delivery.
3. Informed consent: Patients have the right to be informed about the use of AI in their care and to give consent. Healthcare providers must communicate the role of AI in decision-making and obtain informed consent from patients.
4. Accountability and liability: Determining responsibility in case of adverse outcomes or errors resulting from AI-powered decisions can be complex. The legal and ethical frameworks need to address liability issues between the AI developers, healthcare providers, and patients.
5. Transparency and explainability: Healthcare providers and patients should be able to understand how an AI system makes decisions. Transparent and interpretable algorithms are necessary to maintain trust and enable informed decision-making.
6. De-skilling of healthcare professionals: Overreliance on AI could lead to de-skilling of healthcare professionals, making them overly dependent on the technology. It is crucial to strike a balance between AI support and maintaining professional expertise.
7. Human empathy and interaction: AI systems may lack the ability to provide empathy, understanding, and human touch, which are essential aspects of healthcare. The role of AI should complement, not replace, human interaction in patient care.
8. Accessibility and digital divide: AI-driven healthcare advancements should be available to all patients, regardless of socioeconomic background or geographical location. Ensuring access to quality healthcare services without exacerbating existing inequalities is a significant ethical concern.

Addressing these ethical issues requires collaboration among AI developers, healthcare professionals, patients, and regulatory bodies to establish guidelines and best practices that ensure the responsible use of AI in healthcare.

Author’s prompt 2:

How should humans respond to these ethical issues arising from AI?

ChatGPT (GPT-3.5) response 2:

Humans should respond to the ethical issues arising from AI in healthcare by taking a proactive and collaborative approach. Here are some steps that can be taken:

1. Education and awareness: It is essential to educate healthcare providers, patients, and the general public about the ethical issues surrounding AI in healthcare. This will help ensure that everyone is aware of the potential risks and benefits of the technology.
2. Collaboration: The development and deployment of AI in healthcare should involve collaboration between healthcare providers, patients, policymakers, and technology developers. This will help ensure that everyone's perspectives and concerns are taken into account.
3. Ethical guidelines and regulations: There should be clear ethical guidelines and regulations in place to ensure that AI is developed and used in a way that prioritizes patient safety, privacy, and autonomy. These guidelines should be regularly reviewed and updated to keep pace with advancements in technology.
4. Transparency: There should be transparency in the development and use of AI in healthcare. This includes transparency around the data used to train the algorithms, the decision-making process, and the potential biases or limitations of the technology.
5. Accountability: There should be clear accountability for the development and use of AI in healthcare. This includes accountability for any mistakes or harms caused by the technology.

Overall, it is essential to approach the ethical issues arising from AI in healthcare with a focus on collaboration, transparency, and accountability. This will help ensure that the technology is developed and used in a way that benefits patients and society as a whole.

ChatGPT (GPT-4) response 2:

To address the ethical issues arising from AI in healthcare, various stakeholders should take a proactive and collaborative approach. Here are some strategies to consider:

1. Develop clear guidelines and regulations: Governments, regulatory bodies, and professional organizations should work together to develop comprehensive guidelines and regulations governing the use of AI in healthcare. These should encompass data protection, liability, and transparency, among other concerns.
2. Ethical AI design and development: AI developers should prioritize ethical considerations from the onset, following principles such as fairness, transparency, and privacy. Ensuring diverse and representative training data can help minimize biases in AI systems.
3. Encourage interdisciplinary collaboration: AI experts, healthcare professionals, ethicists, social scientists, and patient representatives should collaborate in the development and deployment of AI solutions. This can help address ethical concerns and maximize the benefits of AI in healthcare.
4. Invest in education and training: Healthcare professionals should receive appropriate education and training on AI technologies to understand their potential benefits and limitations. This will enable them to use AI effectively and responsibly in patient care.
5. Promote transparency and explainability: AI developers should work towards creating transparent and interpretable AI systems that allow healthcare providers and patients to understand the decision-making process, fostering trust and informed consent.
6. Engage patients and the public: Patients and the public should be involved in discussions about AI in healthcare to address ethical concerns and ensure that AI technologies are aligned with societal values and patient needs.
7. Foster accessibility and inclusivity: AI-driven healthcare solutions should be designed and deployed in a way that ensures access to quality care for all patients, regardless of socioeconomic status or geographical location.
8. Continuous monitoring and evaluation: Implement regular monitoring and evaluation of AI systems to identify potential biases, errors, or unintended consequences. Adjusting the algorithms or discontinuing certain applications may be necessary based on these assessments.
9. Balance human interaction and AI support: While AI can augment healthcare professionals' abilities, it should not replace the essential human touch and empathy that is a cornerstone of quality care. Striking the right balance is key to harnessing AI's potential without compromising human values.

By adopting these strategies, humans can address the ethical challenges posed by AI in healthcare and ensure that its benefits are maximized while minimizing potential risks and unintended consequences.


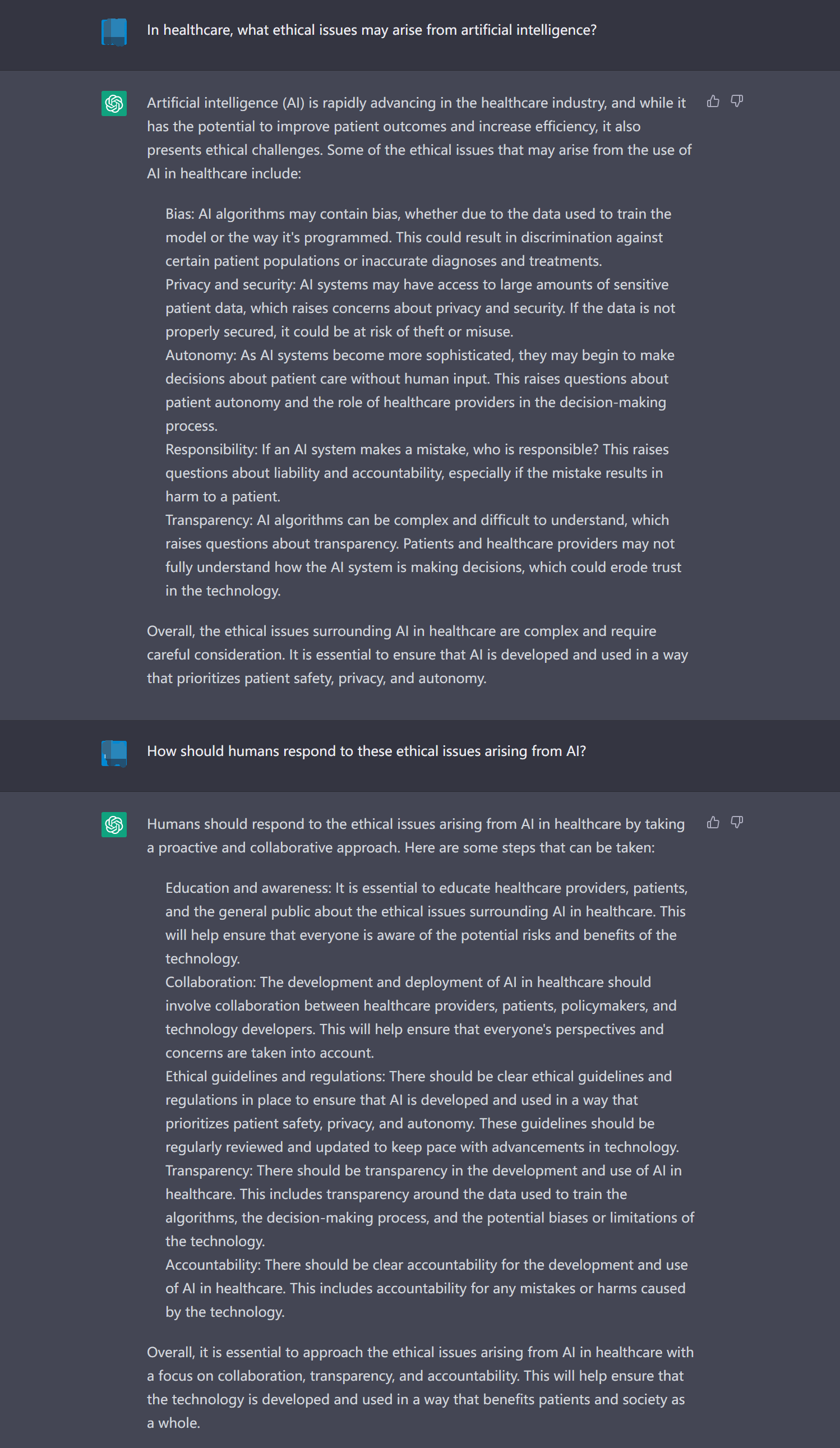


**Task 2**

Author’s prompt 1:

In healthcare, what ethical issues may arise from chatGPT or GPT-4?

ChatGPT (GPT-3.5) response 1:

As an AI language model, chatGPT or future models like GPT-4 may raise various ethical issues in healthcare, including:

1. Privacy and Data Security: ChatGPT may require access to patient data to provide personalized responses, raising concerns about privacy and data security. It is essential to ensure that patient information is protected, and any data collected by the AI model is used only for healthcare purposes.
2. Informed Consent: The use of ChatGPT in healthcare may require obtaining informed consent from patients, particularly in cases where the AI system is making medical decisions on their behalf. Patients must be informed about how the AI system works, the potential benefits and risks, and the alternatives available.
3. Bias and Discrimination: AI systems like ChatGPT may inherit bias from the data they are trained on, which could lead to discrimination against certain groups of patients. It is crucial to ensure that the AI model is trained on diverse and representative datasets and regularly audited to identify and mitigate any biases.
4. Responsibility and Accountability: In cases where ChatGPT makes medical decisions, it raises questions about responsibility and accountability. It is essential to establish clear lines of responsibility, identify who is responsible for the actions taken by the AI system, and ensure that the AI system is transparent in its decision-making process.
5. Medical Ethics: The use of ChatGPT may raise questions about medical ethics, particularly when it comes to issues such as confidentiality, autonomy, and beneficence. It is essential to ensure that the use of AI in healthcare aligns with established ethical principles and guidelines.

Overall, the integration of AI systems like ChatGPT or GPT-4 in healthcare requires careful consideration of the ethical implications and a commitment to upholding ethical standards in healthcare.

ChatGPT (GPT-4) response 1:

In healthcare, several ethical issues may arise from the use of AI-driven technologies like ChatGPT or GPT-4. These include:

1. Data privacy and security: AI models process large volumes of personal and sensitive health information, raising concerns about data privacy and security. Ensuring the protection of patient data and adhering to privacy regulations like HIPAA or GDPR is crucial.
2. Bias and discrimination: AI models trained on historical data can inadvertently learn and perpetuate existing biases present in the data. This can lead to unfair treatment, misdiagnoses, or inappropriate recommendations for underrepresented groups.
3. Transparency and explainability: The 'black-box' nature of AI models can make it difficult to understand how they arrive at their conclusions, leading to a lack of transparency. This poses challenges in terms of trust and accountability when using AI to inform healthcare decisions.
4. Quality and reliability of information: AI-generated content may not always be accurate or relevant, and could be influenced by false information or outdated medical knowledge. Ensuring that AI models are up-to-date and accurate is essential for providing reliable information and advice.
5. Liability and malpractice: It can be challenging to assign responsibility in cases where AI-driven technologies lead to misdiagnoses or inappropriate treatments. Determining the roles of AI developers, healthcare providers, and AI itself in malpractice cases can be complex.
6. Informed consent: Patients may not fully understand the implications of AI-driven healthcare tools or how their data is used. Ensuring that patients are informed about the role of AI in their healthcare and can provide informed consent is an essential ethical consideration.
7. Automation and the doctor-patient relationship: Over-reliance on AI tools can potentially undermine the human connection between doctors and patients, affecting trust, empathy, and overall quality of care. Striking a balance between AI-driven efficiency and maintaining meaningful human interactions is crucial.
8. Access and digital divide: Access to AI-driven healthcare tools may not be equitable, potentially exacerbating existing disparities in healthcare access and outcomes. Addressing the digital divide to ensure equal access to AI tools is an important ethical concern.

Addressing these ethical issues requires a multidisciplinary approach, with collaboration among healthcare professionals, AI developers, policymakers, and ethicists to ensure the responsible and ethical development and deployment of AI-driven healthcare technologies.

Author’s prompt 2:

How should humans respond to the ethical issues arising from chatGPT?

ChatGPT (GPT-3.5) response 2:

Humans must respond to the ethical issues arising from chatGPT and other AI systems in healthcare in a responsible and proactive manner. Here are some ways in which humans can respond to these ethical issues:

1. Establish clear guidelines and standards: Healthcare organizations must establish clear guidelines and standards for the development and deployment of AI systems like chatGPT. These guidelines should cover issues such as privacy, data security, informed consent, bias and discrimination, responsibility and accountability, and medical ethics.
2. Develop transparent decision-making processes: It is essential to develop transparent decision-making processes for the use of chatGPT and other AI systems in healthcare. This includes ensuring that the decision-making process is explainable and that patients can understand how the AI system arrived at its decisions.
3. Monitor and evaluate AI systems regularly: Healthcare organizations must monitor and evaluate AI systems like chatGPT regularly to identify and mitigate any biases or other ethical issues. This includes testing the AI system on diverse datasets and auditing its decision-making process.
4. Educate healthcare professionals and patients: Healthcare professionals and patients must be educated about the ethical issues arising from chatGPT and other AI systems in healthcare. This includes informing patients about how the AI system works, the potential benefits and risks, and the alternatives available.
5. Foster collaboration between different stakeholders: Finally, it is essential to foster collaboration between different stakeholders, including healthcare professionals, patients, AI developers, and policymakers. This collaboration can help ensure that the ethical implications of chatGPT and other AI systems in healthcare are adequately addressed.

Overall, responding to the ethical issues arising from chatGPT and other AI systems in healthcare requires a collaborative and proactive approach from all stakeholders. By working together, we can ensure that the benefits of AI in healthcare are realized while upholding ethical standards and protecting patient rights.

ChatGPT (GPT-4) response 2:

Addressing the ethical issues arising from ChatGPT or other AI-driven technologies requires a proactive and collaborative approach from various stakeholders. Here are some recommendations:

1. Develop robust policies and guidelines: Establish clear, comprehensive, and enforceable policies and guidelines for AI-driven technologies, focusing on privacy, transparency, accountability, and fairness.
2. Encourage multidisciplinary collaboration: Bring together AI developers, ethicists, policymakers, healthcare professionals, and users to discuss and address the ethical concerns. Foster open communication and collaboration among stakeholders.
3. Ensure data privacy and security: Implement strong data protection measures, following privacy regulations such as HIPAA, GDPR, and other relevant laws. Develop techniques to anonymize and protect sensitive information while training AI models.
4. Mitigate bias and discrimination: Encourage the development of diverse, representative datasets for AI training. Implement fairness-aware algorithms and continuously monitor for biased outcomes. Raise awareness about the potential for bias and provide education on ways to address it.
5. Promote transparency and explainability: Develop methods to make AI models more transparent and understandable, such as explainable AI (XAI). Encourage developers to disclose AI system limitations and provide user-friendly explanations of AI-generated content.
6. Establish quality control and continuous improvement: Implement rigorous testing, validation, and auditing of AI-driven technologies to ensure the accuracy, relevance, and reliability of information provided. Keep AI models up-to-date with the latest medical knowledge and best practices.
7. Address liability and malpractice concerns: Develop clear guidelines on the distribution of responsibility and liability among AI developers, healthcare providers, and AI technologies. Establish legal and regulatory frameworks to handle malpractice cases involving AI.
8. Ensure informed consent and patient education: Develop clear consent processes for patients when using AI-driven healthcare tools. Educate patients about the implications of AI in their healthcare and promote awareness of potential risks and benefits.
9. Maintain the human touch in healthcare: Encourage healthcare professionals to strike a balance between using AI-driven tools and maintaining meaningful, empathetic interactions with patients. Emphasize the importance of human judgment and the doctor-patient relationship.
10. Bridge the digital divide: Work towards equitable access to AI-driven healthcare technologies, addressing the digital divide and potential disparities in healthcare outcomes. Encourage the development of low-cost and accessible AI solutions to reach underserved communities.

By adopting these measures, humans can address the ethical issues arising from ChatGPT or similar AI-driven technologies, ensuring their responsible development and deployment in healthcare and other domains.


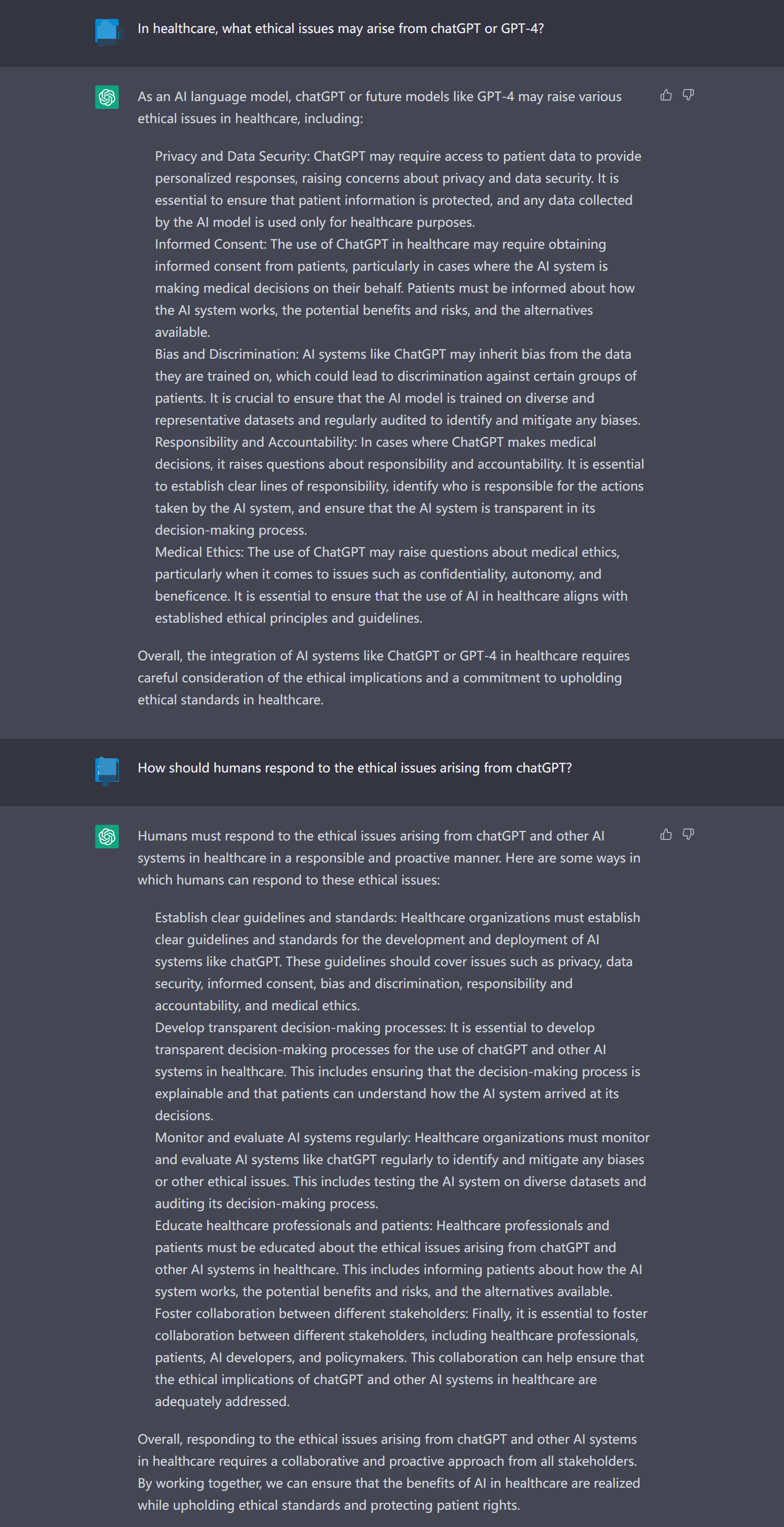

Supplement: Multimedia Appendix 1 [file jmir_v25i1e48009_app1.docx]
